# Emerging trend in second messenger communication and myoendothelial feedback

**DOI:** 10.3389/fphys.2014.00243

**Published:** 2014-06-30

**Authors:** Cam Ha T. Tran, David T. Kurjiaka, Donald G. Welsh

**Affiliations:** ^1^Hotchkiss Brain Institute, University of CalgaryCalgary, AB, Canada; ^2^Libin Cardiovascular Research Institute, University of CalgaryCalgary, AB, Canada; ^3^Department of Physiology and Pharmacology, University of CalgaryCalgary, AB, Canada; ^4^Department of Biomedical Sciences, Grand Valley State UniversityAllendale, MI, USA

**Keywords:** gap junctions, calcium wavelets, constriction, inositol trisphosphate, potassium channels

## Abstract

Over the past decade, second messenger communication has emerged as one of the intriguing topics in the field of vasomotor control. Of particular interest has been the idea of second messenger flux from smooth muscle to endothelium initiating a feedback response that attenuates constriction. Mechanistic details of the precise signaling cascade have until recently remained elusive. In this perspective, we introduce readers to how myoendothelial gap junctions could enable sufficient inositol trisphosphate flux to initiate endothelial Ca^2+^ events that activate Ca^2+^ sensitive K^+^ channels. The resulting hyperpolarizing current would in turn spread back through the same myoendothelial gap junctions to moderate smooth muscle depolarization and constriction. In discussing this defined feedback mechanism, this brief manuscript will stress the importance of microdomains and of discrete cellular signaling.

## Introduction

To optimize blood flow delivery to active tissue, tone in arteriole networks is modified by prevailing mechanical and chemical stimuli. These stimuli affect tone by altering smooth muscle contractility through changes in the phosphorylation state of the 20-kDa regulatory light chain of myosin II (MLC_20_). The proximate regulators of MLC_20_ are myosin light chain- kinase (MLCK) and phosphatase (MLCP), which are in turn controlled by membrane potential (V_M_) and second messenger signaling. When stimuli alter endothelial V_M_, charge moves to smooth muscle through gap junctions (Emerson and Segal, 2000; Berman et al., 2002; de Wit et al., 2006; Haddock et al., 2006) to elicit vasomotor responses (Little et al., 1995; Li and Simard, 2001; Hill et al., 2002). While ionic movement, albeit cations, or anions, through myoendothelial gap junctions (MEGJ) is responsible for the endothelial-dependent hyperpolarization of smooth muscle (Bartlett and Segal, 2000; Emerson and Segal, 2000; Coleman et al., 2001; Budel et al., 2003; Dora et al., 2003; Diep et al., 2005; Domeier and Segal, 2007; Tran et al., 2009), studies have also pointed to the possibility of second messengers flux influencing arterial tone (Dora et al., 1997; Uhrenholt et al., 2007). In this regard, initial work centered on the moderation of vessel constriction through the bulk movement of Ca^2+^ and/or IP_3_ from smooth muscle to endothelium (Dora et al., 1997; Yashiro and Duling, 2000; Lamboley et al., 2005; Isakson et al., 2007). More recently, studies have focused on discrete second messenger movements from smooth muscle to elicit localized Ca^2+^ events in the endothelium (Uhrenholt et al., 2006, 2007; Tallini et al., 2007). This brief review will focus on the nature of second messenger communication and how such movements could elicit “myoendothelial feedback responses.”

## Initial observations of myoendothelial feedback

The functional relevance of myoendothelial feedback was first reported in the context of conducted responses. These vasomotor responses are elicited by discrete agonist-induced changes in V_M_ that travel along the vessel wall (Bartlett and Segal, 2000; Emerson and Segal, 2000; Coleman et al., 2001; Budel et al., 2003; Dora et al., 2003; Diep et al., 2005; Domeier and Segal, 2007; Tran et al., 2009). What intrigued investigators was the inability of smooth muscle agonists, purported to constrict via depolarization, to spread beyond the application site (Dora et al., 1997; Yashiro and Duling, 2000, 2003). This lack of intercellular conduction was attributed to a myoendothelial feedback response that sequentially involved: (1) bulk Ca^2+^ flux across MEGJs from depolarized smooth muscle; (2) global elevation of endothelial [Ca^2+^]; (3) activation of the dilatory effectors (nitric oxide release Dora et al., 1997) or SK/IK channels (Yashiro and Duling, 2000, 2003); (4) redistribution of charge to counter the initial smooth muscle response. While intriguing, recent studies have shown that discrete smooth muscle stimuli fail to elicit conduction due to an inability to initiate depolarization (see Tran et al., 2009; Tran and Welsh, 2009 for details). In light of this finding and a range of biophysical limitations, the vascular field could have dismissed the idea of myoendothelial feedback. Investigators instead revised the concept taking into account new structural information and the ability to measure discrete endothelial Ca^2+^ events.

## Structural composition of myoendothelial contact sites

### Endothelial projections

Resistance arteries are comprised of a single endothelial layer surrounded by one or more smooth muscle layers. The internal elastic lamina (IEL) is a layer of collagen and elastin separating these two cell types. The thickness of the IEL was thought to preclude direct contact between endothelium and smooth muscle. Work over the last decade, however, have revealed the presence of “holes” in the IEL, regions devoid of elastin (Sandow et al., 2002, 2006, 2009; Ledoux et al., 2008b). These regions contain thin endothelial projections that extend through the IEL and make contact with the overlying smooth muscle (Sandow et al., 2002, 2006, 2009). While the process by which they are formed remains elusive, endothelial projections appear to retain structures such as endoplasmic reticulum (ER), caveoli, and trafficking vesicles. More importantly, the proteins essential to controlling resistance vessel tone are preserved. These proteins will be discussed below.

### Gap junctions

Gap junctions are comprised of two docking hemichannels (connexons) that enable the movement of charge (anions and cations) and small metabolites/molecules among neighboring cells (Revel and Karnovsky, 1967). Each connexon is an oligomer of six connexin (Cx) subunits (Caspar et al., 1977; Makowski et al., 1977), each of which possess four hydrophobic membrane-spanning domains, two conserved extracellular domains and three variable intracellular domains. Connexins retain distinct molecular properties and varying connexon composition alters the specific permeability of gap junction channels (Bruzzone et al., 1996; Willecke et al., 2002; Saez et al., 2005). This is exemplified by the ability of Cx40 to enable passive diffusion of IP_3_ a key second messenger (Sneyd et al., 1998; Kanaporis et al., 2011). Among the 21 members of the Cx family, Cx37, Cx40, Cx43, and Cx45 are typically observed in vascular cells (Little et al., 1995; Li and Simard, 2001; Hill et al., 2002). Immunohistochemical evidence suggests that Cx expression in the endothelium is substantively higher than in the smooth muscle (Sandow and Hill, 2000; Sandow et al., 2003). Consistent with this view, coupling resistance among endothelial cells (1.5–3.0 MΩ) (Lidington et al., 2000) was 30 fold lower than among smooth muscle cells (Yamamoto et al., 2001). Interestingly, myoendothelial coupling is orders of magnitude greater than smooth muscle cells (>1800 MΩ) (Yamamoto et al., 2001). This high resistivity is in agreement with the immunohistochemical evidence demonstrating few Cx37 and Cx40 expressed in IEL “holes” (Sandow et al., 2006). Although MEGJs are present in endothelial projections passing through the IEL, not all IEL holes possess endothelial projections. Indeed, as vessel size increases, the incidence of MEGJs appears to decrease (Sandow and Hill, 2000; Sandow et al., 2009) indicative of myoendothelial feedback playing a greater role in small resistance arterioles. As these MEGJs are sparsely distributed, the channels stimulated by transiting second messengers must be very close to MEGJs.

### IP_3_ receptors

The three isoforms of IP_3_R (i.e., IP_3_R1, IP_3_R2, IP_3_R3) are widely expressed and uniquely distributing in a range of cells. In whole mesenteric arteries, all 3 isoforms have been detected, with IP_3_R1 and IP_3_R2 appearing to be heavily expressed in endothelial cells (Ledoux et al., 2008b; Sandow et al., 2009). These receptors are important in vascular tone development, as they are involved in regulating intracellular [Ca^2+^]. IP_3_ binds to the IP_3_Rs and lowers the affinity of the stimulatory site for Ca^2+^, thereby promoting channel opening and release of Ca^2+^ (Bootman et al., 1995; Chalmers et al., 2007). In the presence of IP_3_, these receptors are activated by intracellular [Ca^2+^] of ~300 nM. Functional studies demonstrate that IP_3_Rs on the ER play an important role in myoendothelial feedback as impairing ER Ca^2+^ mobilization and inhibition of IP_3_Rs augmented agonist-induced contraction (Nausch et al., 2012; Tran et al., 2012). The original model for myoendothelial feedback required the flux of second messengers across the MEGJs from the contracting smooth muscle. Given that MEGJ communication is minimal, bulk diffusion of Ca^2+^ alone is unlikely to elevate endothelial [Ca^2+^] (Dora et al., 1997; Kansui et al., 2008). If IP_3_ were to cross the MEGJs to elicit a change in endothelial [Ca^2+^], the IP_3_Rs would have to localize near the myoendothelial contact site in order to elicit a response. Past immunohistochemistry studies support the view that a close spatial relationship between IP_3_Rs and MEGJ proteins (i.e., Cx37 and Cx40) does indeed exist (Ledoux et al., 2008b; Sandow et al., 2009; Nausch et al., 2012; Tran et al., 2012). Localization of IP_3_Rs within the endothelial projections place these receptors in an ideal position to respond when a small quanta of IP_3_ crosses the MEGJs from contracting smooth muscle. Subsequent release of Ca^2+^ from the ER causes a discrete rise in endothelial [Ca^2+^]. In order for a discrete rise in [Ca^2+^] to influence global [Ca^2+^], that Ca^2+^ must be able to affect neighboring Ca^2+^ sensitive ion channels.

### Calcium activated k^+^ channels

The likely candidates for discrete activation by Ca^2+^ are the calcium activated K^+^ channels. Within this family of channels, the SK and IK channels are purported to be the most important in terms of myoendothelial feedback. To date, three members of the SK channel family have been identified (i.e., K_Ca2.1–2.3_). Due to high degree of similarity with other SK channels, the previously identified IK or K_Ca3.1_ channel is often viewed as the fourth member of the SK family. Both K_Ca3.1_ and K_Ca2.3_ channels are predominantly expressed in the endothelial cells (Nilius and Droogmans, 2001; Taylor et al., 2003; Sandow et al., 2006). Both K_Ca2.3_ and K_Ca3.1_ channels lack voltage sensitivity (Ledoux et al., 2008a); they are instead gated by nanomolar intracellular [Ca^2+^] (i.e., EC_50_ 300–500 nM) via coupling of calmodulin to the carboxy-terminus acting as Ca^2+^ sensor (Bond et al., 1999; Schumacher et al., 2001). In order to be involved in myoendothelial feedback, these channels must be localized within endothelial projections where the discrete ER Ca^2+^ release occurs which is also near the MEGJ. In fact, immunohistochemistry has repeatedly shown K_Ca3.1_ channels are expressed in close proximity to MEGJs (Sandow and Hill, 2000; Sandow et al., 2002, 2004, 2006; Haddock et al., 2006; Dora et al., 2008; Tran et al., 2012). However, the K_Ca2.3_ channels appear to be more diffusely distributed (Sandow and Hill, 2000; Sandow et al., 2002, 2006, 2009). Further support for the K_Ca3.1_ channel was the functional evidence showing TRAM34, a K_Ca3.1_ channel blocker, but not apamin, a K_Ca2.x_ channel blocker, inhibit myoendothelial feedback (Nausch et al., 2012; Tran et al., 2012). Thus, the K_Ca3.1_ channel appears to be localized within the endothelial projection where it can be involved in myoendothelial feedback. Activation of endothelial K_Ca3.1_ channels leads to hyperpolarization and mediates relaxation via transmission of hyperpolarizing current through MEGJs.

## Current perspective

**Figure 1:**
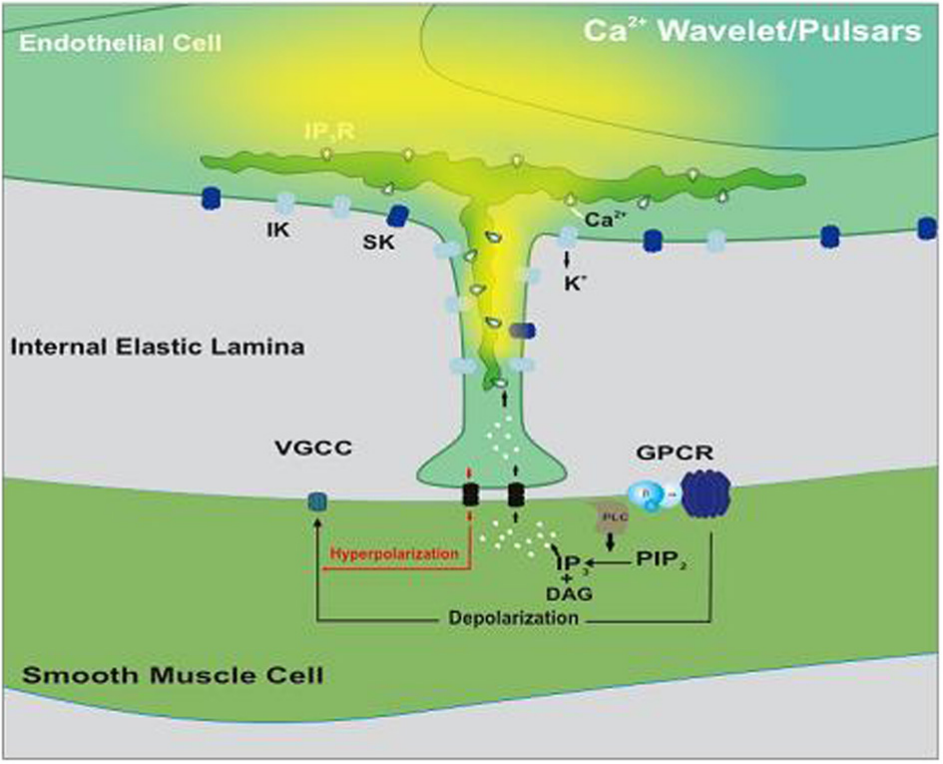
**Illustrative diagram of the myoendothelial feedback pathway**. Smooth muscle agonists activate G protein-coupled receptors (GPCR) initiating IP_3_ production via phospholipase C (PLC). This second messenger crosses myoendothelial gap junctions and triggers Ca^2+^ release via IP_3_Rs positioned on the endoplasmic reticulum. As Ca^2+^ wavelets/pulsars spread, they activate intermediate-conductance Ca^2+^-activated K^+^ (IK) channels within or near the endothelial projection. The resulting hyperpolarization conducts back to smooth muscle where it sequentially attenuates depolarization, Ca^2+^ influx through voltage-gated Ca^2+^ (VGCC) and arterial constriction. Modified from Tran et al. (2012).

The original view of myoendothelial feedback has been adapted and applied to a setting where constrictor agonists are globally applied to induce a depolarization-dependent constriction (Figure 1). The extent of that depolarization, and thereby constriction, is reduced by negative myoendothelial feedback (Tran et al., 2012). This feedback involves the generation of Ca^2+^ wavelets and/or perhaps Ca^2+^ pulsars within or near endothelial projections (Nausch et al., 2012; Tran et al., 2012). Irrespective of whether Ca^2+^ wavelets are kinetically distinct from Ca^2+^ pulsars, both events are spatially and temporally discrete, sensitive to IP_3_R blockade and strikingly distinct from the global elevations of endothelial [Ca^2+^], reported in previous studies (Dora et al., 1997; Yashiro and Duling, 2000; Lamboley et al., 2005). The distinct characteristics of the Ca^2+^ wavelets are consistent with the focal nature of IP_3_R expression within or near the endothelial projections. Local elevations in Ca^2+^ activate K_Ca3.1_ and perhaps K_Ca2.3_ channels expressed near the endothelial projections to elicit hyperpolarization.

## Limitations

Recent observations on myoendothelial feedback have provided mechanistic insights into this process. This perspective is, however, built on measurements that assess the outcome of second messenger flux and not transcellular flux itself. This is due to the absence of techniques to directly evaluate IP_3_ movement. It should also be recognized that the structural requisites for myoendothelial feedback might not be present in all resistance arteries. As such, caution should be applied when extending current findings beyond the vascular beds of skeletal muscle or the mesentery.

## Conclusions

In summary, our understanding of the role myoendothelial feedback plays in vascular function has undergone considerable refinement over the past decade. Starting from the unlikely model of bulk Ca^2+^ flux (Dora et al., 1997; Yashiro and Duling, 2000, 2003), the field has progressed to a more discrete model involving specific channels and receptors positioned in close proximity to one another (Tran et al., 2012). The discrete character of this response was highlighted herein to provide a framework to evaluate other vascular functions that might be impacted by myoendothelial feedback (i.e., angiogenesis). At the same time, this work has implications for our understanding of vascular pathologies like hypertension where conduction along the endothelium is reduced (Kurjiaka, 2004; Kurjiaka et al., 2005). As conduction relies on communication through MEGJs, this apparent decline in MEGJ might be accompanied by a reduction in myoendothelial feedback, which could contribute to the increased constriction observed in the hypertensive vasculature. In any case, further work is required to better understand the functional implications of myoendothelial feedback for the resistance vasculature.

### Conflict of interest statement

The authors declare that the research was conducted in the absence of any commercial or financial relationships that could be construed as a potential conflict of interest.
